# Chromosome Segregation–1–like Gene Participates in Ferroptosis in Human Ovarian Granulosa Cells via Nucleocytoplasmic Transport

**DOI:** 10.3390/antiox13080911

**Published:** 2024-07-28

**Authors:** Luanqian Hu, Tongtong Hong, Yuheng He, Huiyuan Wang, Jinxiang Cao, Danhua Pu, Li Gao, Chao Gao, Yugui Cui, Jie Wu, Rongrong Tan

**Affiliations:** 1 Department of Obstetrics and Gynecology, The First Affiliated Hospital of Nanjing Medical University, Nanjing 210000, China; huluanqian@stu.njmu.edu.cn (L.H.); hongtongtong@stu.njmu.edu.cn (T.H.); heyuheng26@stu.njmu.edu.cn (Y.H.); wanghy@njmu.edu.cn (H.W.); caocaojinxiang@stu.njmu.edu.cn (J.C.); pudanhua@njmu.edu.cn (D.P.); gaoli_gao@126.com (L.G.); gaochao@jsph.org.cn (C.G.); cuiygnj@njmu.edu.cn (Y.C.); 2 State Key Laboratory of Reproductive Medicine and Offspring Health, Nanjing Medical University, Nanjing 210000, China

**Keywords:** CSE1L, nucleocytoplasmic transport, ferritinophagy, ferroptosis, premature ovarian insufficiency

## Abstract

Premature ovarian insufficiency (POI) is defined as the depletion of ovarian function before the age of 40 years. The global prevalence of POI is 3.5%. To date, genetic factors account for 23.5% of the etiology of POI. Herein, a previously uncharacterized pathogenic homozygous variant of the chromosome segregation–1–like gene (CSE1L) was identified in POI patients via targeted panel sequencing. It is reported that dysregulated iron metabolism is involved in many reproductive endocrine disorders; however, its precise role in POI remains obscure. In this study, we identified CSE1L as a potential candidate gene that plays an important role in maintaining iron homeostasis. Deficiency of CSE1L led to ferroptosis in human granulosa cells, which was confirmed by transmission electron microscopy. Mechanistically, coimmunoprecipitation identified the direct interaction between CSE1L and FoxO1. Inhibition of CSE1L led to the excessive accumulation of FoxO1 in the nucleus via nucleocytoplasmic transport. Then, FoxO1 bound to the promoter region of NCOA4 and promoted its transcription, which was verified by a chromatin immunoprecipitation assay. Moreover, inhibition of CSE1L in cumulus cell monolayer could impede oocyte maturation, which might be associated with oxidative stress. Consequently, our study first revealed that CSE1L participated in ferroptosis in human ovarian granulosa cells via nucleocytoplasmic transportation, which might be helpful in revealing the molecular mechanism of CSE1L in the development of POI. Importantly, these findings might provide new insights into the application of ferroptosis inhibitors in the treatment of POI.

## 1. Introduction

Premature ovarian insufficiency (POI) is defined as oligo/amenorrhea for at least 4 months and an elevated FSH level > 25 IU/L on two occasions > 4 weeks apart [1]. Besides menstrual disorders and infertility, POI also leads to cardiovascular disease and osteoporosis, which might pose a threat to women’s health [2]. What is more, the decline of ovarian function is irreversible, and there is currently no effective method to restore ovarian function [2]. Therefore, early diagnosis of POI is of great significance.

Recently, with the rapid development of next–generation sequencing (NGS), more and more causative genes of POI have been identified [3]. Herein, CSE1L was first identified in POI patients via targeted panel sequencing. Of note, CSE1L plays an indispensable role in maintaining cellular homeostasis via nucleocytoplasmic transportation [4]. For example, Nagashima et al. [5] found that CSE1L could enhance malignancy in cancer cells by promoting the nuclear translocation of TAZ. Dong et al. [6] found that CSE1L was involved in epigenetic silencing via nucleocytoplasmic transport. Importantly, it has been reported that nucleocytoplasmic transportation is involved in aging–related neurodegenerative diseases [7]. However, the role of nucleocytoplasmic transportation in POI has not been revealed. Moreover, CSE1L is involved in many biological processes, such as apoptosis, ferroptosis, epigenetic silencing, embryonic development, and so on [4,6,8,9,10,11]. In 2001, Bera et al. [11] identified an embryonically lethal phenotype in homozygous CSE1L–deficiency mice, indicating that CSE1L was essential to embryonic development. However, the precise mechanism is unknown. In addition, recent studies have revealed that CSE1L is crucial to spermatogenesis [12,13]. For example, Liu et al. [12] found that silencing CSE1L in TCam–2 cells led to a multipolar spindle. Although CSE1L has been confirmed to be essential to spermatogenesis and embryonic development, the pathogenicity of CSE1L in POI remains obscure. Given that CSE1L is involved in ferroptosis in NSCLC cells [8], we speculated that CSE1L might participate in the development of POI via ferroptosis.

It is widely reported that iron overload can induce a Fenton reaction, thus increasing the sensitivity to ferroptosis [14]. The homeostasis of iron largely depends on the balance of iron uptake, storage, and efflux [15]. Recently, more and more studies have illustrated that ferroptosis is involved in many reproductive endocrine disorders, including endometriosis, polycystic ovary syndrome, preeclampsia, and so on [16]. A clinical study found that the serum ferrition levels were higher in PCOS patients compared with normal women, indicating that iron overload existed in PCOS patients [17]. Mechanistically, compensatory hyperinsulinemia induced by PCOS could promote iron uptake and inhibit iron release [18,19]. Zhang et al. [20] found that fetal loss in PCOS pregnant rats might be associated with ferroptosis. Li et al. [21] found that iron overload existed in the peritoneal fluid, peritoneal macrophages, and ectopic lesions of endometriosis patients. Further, an oxidative stress microenvironment in the pelvis induced by iron overload could damage mesothelial cells and promote the proliferation, migration, and angiogenesis of ectopic endothelial cells, thus participating in the development of endometriosis [21]. Moreover, the persistent inflammatory response induced by oxidative stress in the pelvis of endometriosis patients has a toxic effect on embryo development [16]. For example, embryos treated with peritoneal fluid from patients with endometriosis exhibited reduced GPX4 expression and increased lipid peroxidation, leading to abnormal early embryonic development [22]. Primate spatiotemporal transcriptomic atlas suggested that genes related to oxidative stress, lipid metabolism, and autophagy were enriched in aging ovaries [23]. Similarly, Jia et al. [24] found that genes related to ferroptosis were significantly enriched in granulosa cells extracted from aging ovaries. In 2022, Wang et al. [25] demonstrated that the loss of BNC1 triggered ferroptosis in oocytes, which first revealed the role of ferroptosis in POI. Although some studies suggest that ferroptosis is closely related to ovarian function, very little is currently known about the role of ferroptosis in POI.

Herein, a previously uncharacterized pathogenic homozygous variant of CSE1L was identified in POI patients. We found that mutant CSE1L (c. 1261T > C) inhibited the viability of human granulosa cells. To investigate the mechanism, RAN–seq was performed, and the results showed that CSE1L was closely related to iron metabolism. Mechanistically, the inhibition of CSE1L resulted in the excessive accumulation of FoxO1 in the nucleus. Then, transcription factor FoxO1 bound to the promoter region of NCOA4 and subsequently promoted the transcription of NCOA4, which would further induce ferritinophagy and increase the sensitivity to ferroptosis. Moreover, a co–culture system of cumulus cell monolayers and denuded oocytes was constructed to evaluate the development of oocytes. The results showed that the inhibition of CSE1L in cumulus cell monolayers could impede oocyte maturation via oxidative stress. Together, our study first demonstrated that the inhibition of CSE1L could induce ferroptosis in human granulosa cells via nucleocytoplasmic transportation, which might be helpful in revealing the molecular mechanisms of CSE1L in the pathogenesis of POI.

## 2. Materials and Methods

### 2.1. Targeted Panel Sequencing

Peripheral blood extracted from 150 POI patients and 150 healthy women was sent to Kaiumph Medical Diagnostics (Beijing, China) for targeted panel sequencing. All subjects signed informed consent forms. The human study was approved by the First Affiliated Hospital of Nanjing Medical University (Approval code: 2013–MD–062).

### 2.2. Animals and Ethics Statement

C57BL/6 mice were purchased from Weitong Lihua Limited Company (Beijing, China). The mice were housed in cages at a temperature range of 24 °C–26 °C under a 12:12 h light–dark cycle with free access to standard food and water. Animal procedures were approved by the Institutional Animal Care and Use Committee (IACUC) of Nanjing Medical University (no. IACUC–2309037).

### 2.3. Cell Culture

Generally speaking, there are many ovarian granulosa cell lines, such as KGN, SVOG, and COV434 cells. Among them, KGN and SVOG cells are widely recognized and used in experiments. Therefore, we chose KGN and SVOG cells as in vitro models. Moreover, dual–model verification can increase the reliability of this study. KGN and SVOG were obtained from the State Key Laboratory of Reproductive Medicine of Nanjing Medical University. Cells were cultured in DMEM medium or RPMI 1640 medium (Zhongqiao Xinzhou Biotechnology Co., Shanghai, China) supplemented with 10% fetal bovine serum (Yeasen BioTechnologies co., Shanghai, China) and 1% penicillin/streptomycin (Gibco) at 37 °C under a humidified atmosphere of 5% CO_2_.

### 2.4. Transfection

Short hairpin RNA against CSE1L (sh–CSE1L), CSE1L overexpression of the lentivirus, and negative control were transfected into human granulosa cells by Corues Biotechnology (Nanjing, China). The RNA sequence of sh–CSE1L is listed in Table S1.

### 2.5. RAN–Seq and Analysis

The total RNA was extracted using an RNA–Quick extraction kit (ES Science, Shanghai, China). Then, the RNA sample was sent to BGI (BGI, Wuhan, China) for RNA–seq and bioinformatics analysis.

### 2.6. Immunofluorescence

For cell preparation, a round coverslip was plated into each well of twelve–well plates, and then the cells were seeded evenly across the wells. The coverslips were fixed, permeated, and blocked in accordance with standard protocol after the cells were 80% confluent. For tissue preparation, ovaries collected from 8–week–old C57BL/6 mice were fixed with 4% paraformaldehyde and embedded in paraffin, followed by a tissue section. Slides were gradient–dewaxed, rehydrated, antigen–repaired, and blocked according to the standard protocol. Then, the round coverslips and slides were incubated with primary antibodies at 4 °C overnight and secondary antibodies in the darkness at room temperature for 1 h the next day. Nuclei were counterstained with Hochest. Immunofluorescence signals were observed by confocal microscopy (Nikon, Tokyo, Japan). Antibodies used in this study are shown in Table S2.

### 2.7. Cell Viability Assessment

Cell viability was examined by a Cell Counting Kit–8 (APExBIO, Houston, TX USA) according to the manufacturer’s protocol. The absorbance at 450 nm was measured by a microplate reader (MuLTiSKAN, Thermo, Waltham, MA, USA).

### 2.8. Reactive Oxygen Species Assessment

Cells were treated with DCFH–DA (Beyotime, Shanghai, China) according to the manufacturer’s protocol. Fluorescence at 488/525 nm was detected by a fluorescence microplate.

### 2.9. Malonaldehyde Assessment

Lipid peroxidation levels in human granulosa cells were examined using an MDA assay kit (Beyotime) in accordance with the manufacturer’s protocol. Fluorescence at 532 nm was detected by a microplate reader (MuLTiSKAN, Thermo, MA, USA). MDA levels were examined by the ratio of absorbance at 532 nm to the protein concentration.

### 2.10. Ferrous Iron Detection

The ferrous iron concentration in human granulosa cells was detected by a Ferrous Ion Content Assay Kit (Beijing Boxbio Science & Technology Co., Ltd., Beijing, China). Then, the ferrous iron concentration was examined according to the formula described in the manufacturer’s protocol.

### 2.11. Transmission Electron Microscopy

Cells harvested by centrifugation at 1000 rpm for 5 min were sent to the Analysis and Testing Center of Nanjing Medical University (Nanjing, China) in the ultrathin section. Then, the ultrathin sections were examined using transmission electron microscopy (JEOL JEM–1400 Flash, Tokyo, Japan).

### 2.12. RNA Extraction and qRT–PCR Assay

An RNA–Quick extraction kit (ES Science, Shanghai, China) was used for total RNA extraction. Then, cDNA was synthesized through a PrimeScript RT Reagent Kit (Vazyme, Nanjing, China). Finally, qRT–PCR was performed using a Taq Pro Universal SYBR qPCR Master Mix (Vazyme, Nanjing, China). The sequences of the primers are shown in Table S3.

### 2.13. Western Blot

Cells were lysed with RIPA lysis buffer (Beyotime, Shanghai, China) supplemented with proteinase inhibitors (Beyotime, Shanghai, China). The supernatant was collected after being centrifuged with 12,000 r for 15 min at 4 °C. Then, a BCA Protein Assay Kit (Yeasen BioTechnologies co., Shanghai, China) was used to examine the protein concentration. A total of 40 µg of the protein sample was loaded into sodium dodecyl sulfate polyacrylamide gel electrophoresis (SDS–PAGE). Separated proteins were transferred to the PVDF membrane. The PVDF membrane was blocked with 5% non–fat milk in TBST for 1 h. After being incubated with primary antibodies and secondary antibodies, the visualization of protein bands was achieved by an ECL Chemiluminescent Substrate Reagent Kit (Yeasen BioTechnologies Co., Shanghai, China). Antibodies used in this study are listed in Table S2.

### 2.14. Chromatin Immunoprecipitation Assay

ChIP assay was conducted using a ChIP assay kit (Beyotime, Shanghai, China) according to the manufacturer’s protocol. First, human granulosa cells were cross–linked with formaldehyde. Then, chromatin fragmentation was achieved by an ultrasound. The prepared chromatin solution was incubated with the FoxO1 antibody and IgG antibody, respectively. Afterward, the above solution was incubated with protein A + G agarose beads. Then, the eluted DNA was collected for PCR after washing. The primers used in the PCR are shown in Table S3.

### 2.15. Coimmunoprecipitation

Cells were lysed with an NP40 lysis buffer (Beyotime, Shanghai, China) supplemented with proteinase inhibitors. The supernatant was collected after being centrifuged with 12,000 r for 15 min at 4 °C. Then, the collected supernatant was incubated with specific antibody–bound–protein A/G magnetic beads (MCE, Monmouth Junction, NJ, USA) overnight. Then, samples were eluted by SDS loading buffer for Western blotting.

### 2.16. Cumulus Cell Monolayer–Denuded Oocyte In Vitro Co–Culture

**Figure 1:**
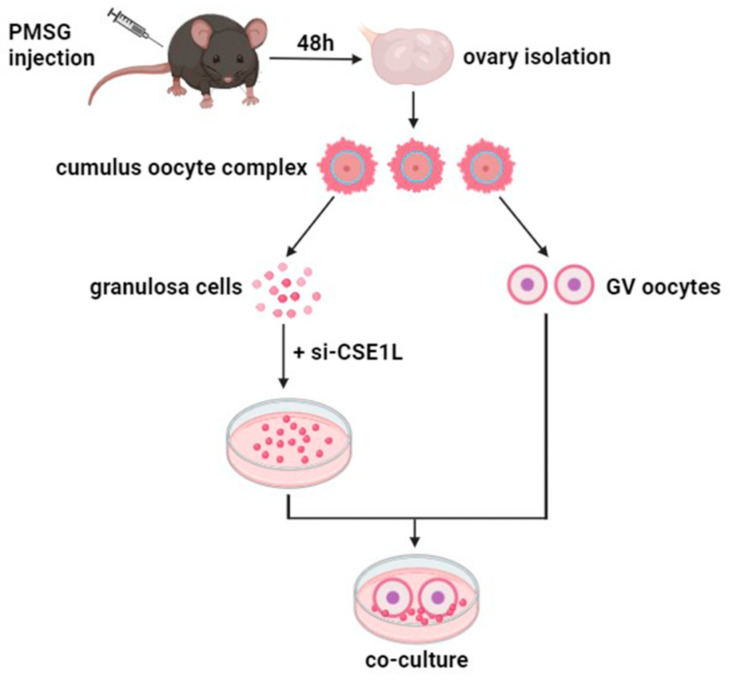
The sketch of co–culture system of cumulus cell monolayer and denuded oocyte.

Three–week–old C57BL/6 mice were intraperitoneally injected with 5 IU pregnant mare’s serum gonadotropin (Ningbo A Second Hormone Factory, Ningbo, China) 46–48 h prior to ovary collection. Then, the ovaries were isolated and placed in an M2 medium (Nanjing Aibei Biotechnology Co., Nanjing, China). Cumulus oocyte complexes (COC) were released from the ovary by puncturing via a hypodermic needle. Cumulus cells mechanically denuded from COC were cultured in vitro to obtain cumulus cell monolayers (CCMs). Then, a co–culture system of cumulus cell monolayers and denuded oocytes was constructed for further experiments (Figure 1).

### 2.17. Statistical Analysis

Statistical analysis was performed using the GraphPad Prism 9 software (GraphPad Software 9.0, Boston, MA, USA). Normality was examined using the Shapiro–Wilk test. For normally distributed data, parametric test was used. Otherwise, nonparametric test was performed. Comparisons between the two groups were analyzed by unpaired Student’s *t*-test if the data satisfied homogeneity of variance. Otherwise, Student’s *t*-test with Welch’s correction was used. Comparisons between more than two groups were analyzed by one–way ANOVA test and two–way ANOVA test if the data satisfied homogeneity of variance. Otherwise, Brown–Forsythe and Welch ANOVA test was used. All experiments were repeated at least three times unless otherwise stated. Normally distributed data were expressed as the mean ± standard deviation. Values of *p* < 0.05 were considered as statistically significant.

## 3. Results

### 3.1. Pathogenetic Variant of CSE1L Identified in POI Patients

**Figure 2:**
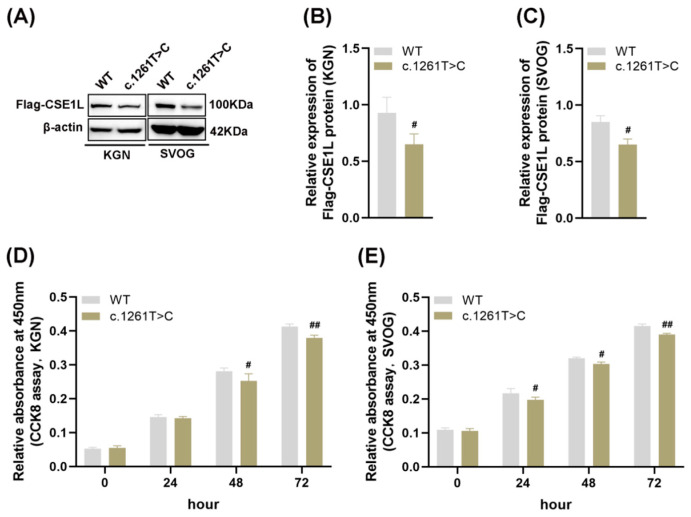
Mutant CSE1L (c. 1261T>C) inhibited the viability of human granulosa cells. (**A**) Flag–CSE1L protein expression in KGN and SVOG cells transfected with mutant CSE1L (c. 1261T>C) plasmid was detected by Western blotting. (**B**,**C**) Quantification of band intensity of Flag–CSE1L/β–actin ratios in KGN and SVOG cells. (**D**,**E**) Cell viability in KGN and SVOG cells was detected by CCK8 assay. N = 3 for each group. Data were expressed as mean ± SD. ^#^ *p* < 0.05 compared to WT, ^##^ *p* < 0.01 compared to WT.

Four–point mutations (NM_001256135: c. 1042A>G, p. I348V; c. 1261T>C, p. F421L; c. 1264C>T, p. P422S; c. 409C>T, p. R137C) and one splicing mutation (NM_001256135: c. 477–3T>C) in CSE1L were identified in POI patients. Then, AlphaFold2, an artificial intelligence system developed by DeepMind, was used to predict the protein structure [26]. The results showed that compared with other mutations, c. 1261T>C might lead to protein structural instability (Table S4). As shown in Figure 2A–C, mutant CSE1L (c. 1261T>C) plasmid decreased the expression of CSE1L in human granulosa cells. Moreover, cell viability was significantly inhibited after transfecting mutant CSE1L (c. 1261T>C) plasmid (Figure 2D,E).

### 3.2. The Expression of CSE1L was Downregulated in Aged Mice Ovaries

**Figure 3:**
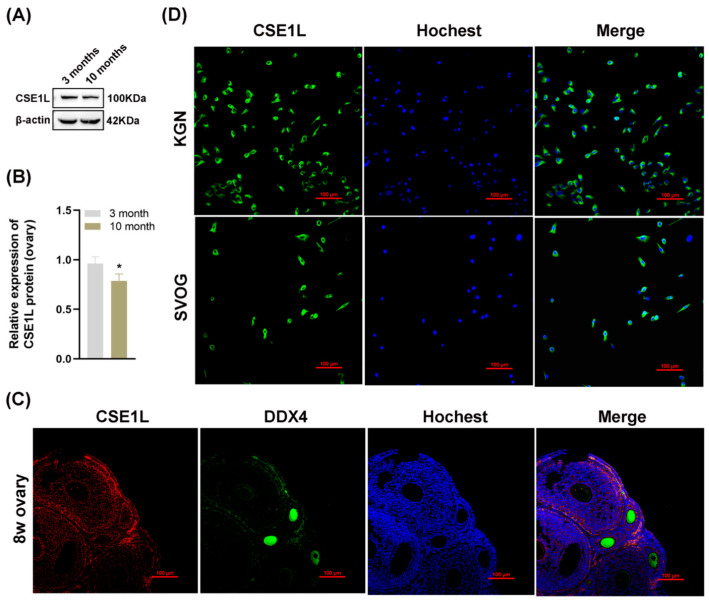
The expression of CSE1L was downregulated in aged mice ovaries. (**A**) CSE1L protein expression in ovaries was detected by Western blotting. (**B**) Quantification of band intensity of CSE1L/β–actin ratios in ovaries. (**C**) Localization of CSE1L in the 8w ovary. Immunofluorescence staining of CSE1L (red), DDX4 (green) in ovary were analyzed by confocal microscopy. Nuclei were counterstained with Hochest (blue). Scale bar: 100 µm. (**D**) Cell localization of CSE1L in KGN and SVOG cell. Immunofluorescence staining of CSE1L (green), Nuclei (blue) in cells were analyzed by confocal microscopy. Scale bar: 100 µm. N = 3 for each group. Data were expressed as mean ± SD. * *p* < 0.05 compared to 3–month–old mice.

To further investigate the role of CSE1L in ovarian function, we examined the expression of CSE1L in ovaries obtained from young and old mice. As shown in Figure 3A,B, the expression of CSE1L was decreased in ovaries obtained from old mice. In addition, immunofluorescence suggested that CSE1L was expressed in granulosa cells and oocytes in mice ovaries (Figure 3C). In human granulosa cells, CSE1L was mainly located in the cytoplasm (Figure 3D). Thus, we believed that CSE1L was crucial to ovarian function.

### 3.3. CSE1L Silence–Affected Iron Metabolism in Human Ovarian Granulosa Cells

**Figure 4:**
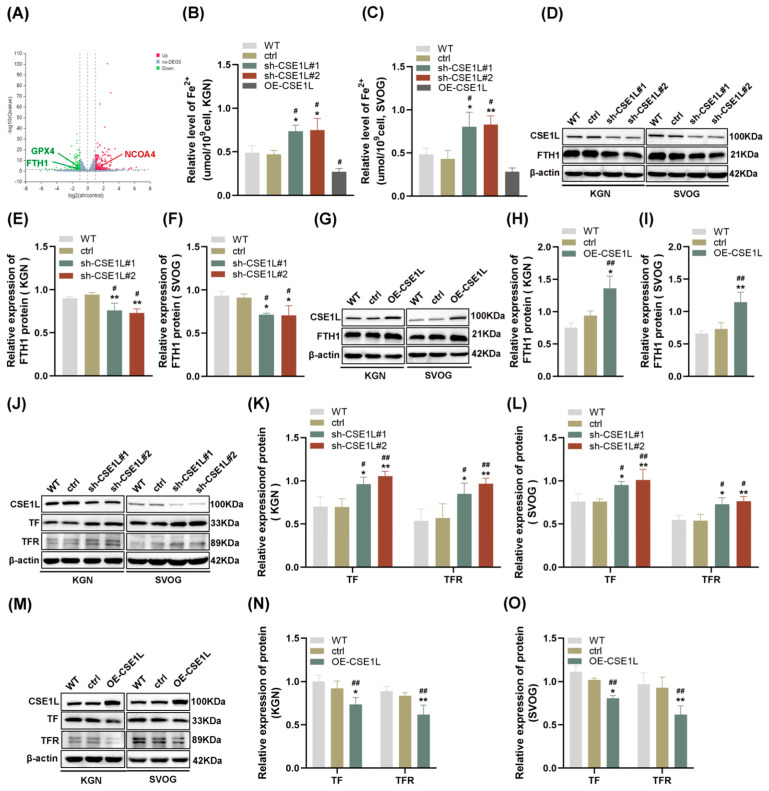
CSE1L silence affected iron metabolism in human granulosa cells. (**A**) Volcano plot of differentially expressed genes (Q value ≤ 0.05, |log2FC| ≥ 1). (**B**,**C**) Ferrous iron level in KGN and SVOG cells transfected with sh–CSE1L or CSE1L overexpression of the lentivirus. (**D**) FTH1 protein expression in KGN and SVOG cells transfected with sh–CSE1L was detected by Western blotting. (**E**,**F**) Quantification of band intensity of FTH1/β–actin ratios in KGN and SVOG cells transfected with sh–CSE1L. (**G**) FTH1 protein expression in KGN and SVOG cells transfected with CSE1L overexpression of the lentivirus was detected by Western blotting. (**H**,**I**) Quantification of band intensity of FTH1/β–actin ratios in KGN and SVOG cells transfected with CSE1L overexpression of the lentivirus. (**J**) TF, TFR protein expression in KGN and SVOG cells transfected with sh–CSE1L was detected by Western blotting. (**K**,**L**) Quantification of band intensity of TF/β–actin, TFR/β–actin ratios in KGN and SVOG cells transfected with sh–CSE1L. (**M**) TF, TFR protein expression in KGN and SVOG cells transfected with CSE1L overexpression of the lentivirus was detected by Western blotting. (**N**,**O**) Quantification of band intensity of TF/β–actin, TFR/β–actin ratios in KGN and SVOG cells transfected with CSE1L overexpression of the lentivirus. N = 3 for each group. Data were expressed as mean ± SD. ^#^ *p* < 0.05 compared to WT, ^##^ *p* < 0.01 compared to WT. * *p* < 0.05 compared to ctrl, ** *p* < 0.01 compared to ctrl.

To further explore the effect of CSE1L on human granulosa cells, CSE1L stable knockdown cell lines were constructed by two different shRNAs, respectively. Both of the two different shRNAs could significantly downregulate the expression of CSE1L in KGN and SVOG cells (Figure S1). Then, RNA–seq was performed. The results showed that 163 genes were upregulated and 110 genes were downregulated after silencing CSE1L in KGN cells (Q value ≤ 0.05, |log2FC| ≥ 1) (Figure 4A). Among them, iron–metabolism–associated genes were included. Then, the ferrous iron level in human granulosa cells transfected with sh–CSE1L was assessed. The results showed that contrary to CSE1L overexpression of the lentivirus, sh–CSE1L could significantly upregulate ferrous iron levels in human granulosa cells (Figure 4B,C). To further investigate the mechanism of abnormal ferrous iron levels, proteins involved in iron storage (FTH1) and iron take up (TF, TFR) were examined. As expected, sh–CSE1L significantly upregulated the expression of TF and TFR and downregulated the expression of FTH1 in human granulosa cells compared to CSE1L overexpression of the lentivirus (Figure 4D–O). Thus, we can conclude that loss of CSE1L induce an intracellular iron overload by promoting iron uptake and destroying iron storage.

### 3.4. Intracellular Iron Overload Induced by CSE1L Silence Promoted Ferroptosis in Human Ovarian Granulosa Cells

**Figure 5:**
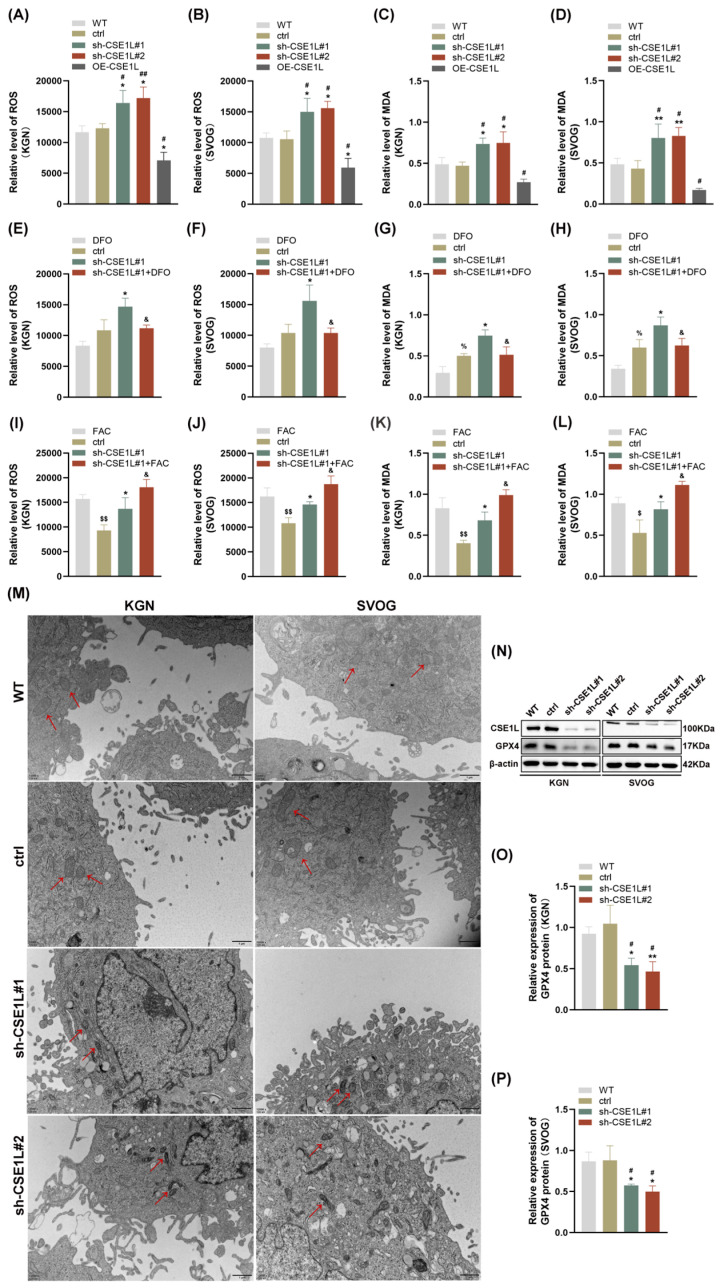
CSE1L silence induced ferroptosis in human granulosa cells. (**A**,**B**) ROS level in KGN and SVOG cells transfected with sh–CSE1L or CSE1L overexpression of the lentivirus. (**C**,**D**) MDA level in KGN and SVOG cells transfected with sh–CSE1L or CSE1L overexpression of the lentivirus. (**E**–**H**) ROS and MDA level in KGN and SVOG cells treated with DFO. (**I**–**L**) ROS and MDA level in KGN and SVOG cells treated with FAC. (**M**) Images from TEM showing morphology of mitochondria (red arrows) in human granulosa cells transfected with sh–CSE1L. Scale bar: 1 µm (**N**) GPX4 protein expression in KGN and SVOG cells transfected with sh–CSE1L was detected by Western blotting. (**O**,**P**) Quantification of band intensity of GPX4/β–actin ratios in KGN and SVOG cells transfected with sh–CSE1L. N = 3 for each group. Data were expressed as mean ± SD. ^#^ *p* < 0.05 compared to WT, ^##^ *p* < 0.01 compared to WT. * *p* < 0.05 compared to ctrl, ** *p* < 0.01 compared to ctrl. ^%^ *p* < 0.05 compared to cells treated with DFO. ^$^ *p* < 0.05 compared to cells treated with FAC, ^$$^ *p* < 0.01 compared to cells treated with FAC. ^&^ *p* < 0.05 compared to cells transfected with sh–CSE1L#1.

As we all know, disruption of iron homeostasis can lead to an intracellular iron overload, which further catalyzes hydrogen peroxide into superoxide radicals and hydroxyl radicals, thus triggering oxidative stress and lipid peroxidation [27]. This biological process is defined as the Fenton reaction [27]. Then, ROS and MDA levels were examined in human granulosa cells. As shown in Figure 5A–D, contrary to CSE1L overexpression of the lentivirus, sh–CSE1L significantly upregulated ROS and MDA levels, indicating that oxidative stress and lipid peroxidation were activated. To further investigate whether the oxidative stress and lipid peroxidation were dependent on the ferrous iron level, we used DFO to chelate intracellular ferrous iron. Additionally, FAC was used to supply exogenous iron. As shown in Figure 5E–L, DFO could partially alleviate the oxidative stress and lipid peroxidation induced by sh–CSE1L, while FAC had the opposite effect. A transmission electron microscopy (TEM) assay suggested that mitochondria in cells transfected with sh–CSE1L showed increased membrane density and shrunken morphology (Figure 5M). Moreover, we found that sh–CSE1L significantly decreased the expression of GPX4 (Figure 5N–P). In contrast, CSE1L overexpression of the lentivirus increased the expression of GPX4 (Figure S2). Taken together, CSE1L might play an essential role in ferroptosis.

### 3.5. CSE1L Silence Promoted Ferroptosis via Activating NCOA4–Mediated Ferritinophagy

**Figure 6:**
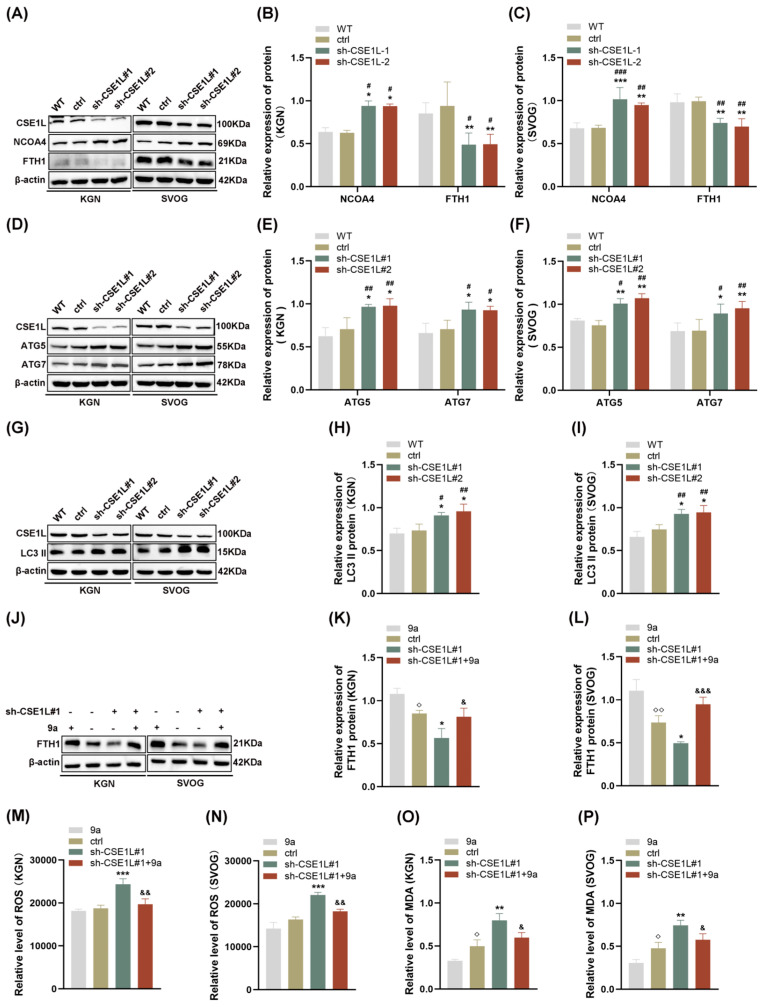
CSE1L silence promoted ferroptosis via activating NCOA4–mediated ferritinophagy. (**A**) NCOA4, FTH1 protein expression in KGN and SVOG cells transfected with sh–CSE1L was detected by Western blotting. (**B**,**C**) Quantification of band intensity of NCOA4/β–actin, FTH1/β–actin ratios in KGN and SVOG cells transfected with sh–CSE1L. (**D**) ATG5, ATG7 protein expression in KGN and SVOG cells transfected with sh–CSE1L was detected by Western blotting. (**E**,**F**) Quantification of band intensity of ATG5/β–actin, ATG7/β–actin ratios in KGN and SVOG cells transfected with sh–CSE1L. (**G**) LC3 II protein expression in KGN and SVOG cells transfected with sh–CSE1L was detected by Western blotting. (**H**,**I**) Quantification of band intensity of LC3 II/β–actin ratios in KGN and SVOG cells transfected with sh–CSE1L. (**J**) FTH1 protein expression in KGN and SVOG cells treated with 9a was detected by Western blotting. (**K**,**L**) Quantification of band intensity of FTH1/β–actin ratios in KGN and SVOG cells treated with 9a. (**M**–**P**) ROS and MDA level in KGN and SVOG cells treated with 9a. N = 3 for each group. Data were expressed as mean ± SD. ^#^ *p* < 0.05 compared to WT, ^##^ *p* < 0.01 compared to WT, ^###^ *p* < 0.001 compared to WT. * *p* < 0.05 compared to ctrl, ** *p* < 0.01 compared to ctrl, *** *p* < 0.001 compared to ctrl. ^◇^ *p* < 0.05 compared to cells treated with 9a, ^◇◇^ *p* < 0.01 compared to cells treated with 9a. ^&^ *p* < 0.05 compared to cells transfected with sh–CSE1L#1, ^&&^ *p* < 0.01 compared to cells transfected with sh–CSE1L#1, ^&&&^ *p* < 0.001 compared to cells transfected with sh–CSE1L#1.

Autophagy, famous as a conserved degradation pathway, is essential for maintaining cellular homeostasis [28]. However, excessive autophagy is lethal to cells. Growing evidence confirms that autophagy plays an important role in gonad development and gametogenesis [29]. In 2022, Amrita et al. [9] first reported that the loss of CSE1L could increase the expression of LC3B–II, indicating that CSE1L participated in autophagy. In our study, we found that sh–CSE1L significantly upregulated the expression of NCOA4 and downregulated the expression of FTH1 (Figure 6A–C), which was consistent with RNA–seq. Moreover, the expression of ATG5, ATG7, and LC3 II was increased in human granulosa cells transfected with sh–CSE1L, indicating that a large number of autophagosomes were formed (Figure 6D–I). Overall, NCOA4 bound to FTH1 and subsequently delivered FTH1 to the lysosome via autophagosome in the presence of LC3 and ATG5/ATG7. Fang et al. [30] found that compound 9a could disrupt NCOA4–FTH1 interaction at 0.5 µM, thus inhibiting ferritinophagy. Herein, we found that compound 9a partially alleviated the decreased FTH1 induced by sh–CSE1L (Figure 6J–L). Further, ROS and MDA levels were examined, and the results showed that compound 9a partially alleviated the oxidative stress and lipid peroxidation induced by sh–CSE1L (Figure 6M–P). Together, the data suggest that sh–CSE1L promoted ferroptosis via activating NCOA4–mediated ferritinophagy.

### 3.6. CSE1L Participated in Ferritinophagy via Nucleocytoplasmic Transport

**Figure 7:**
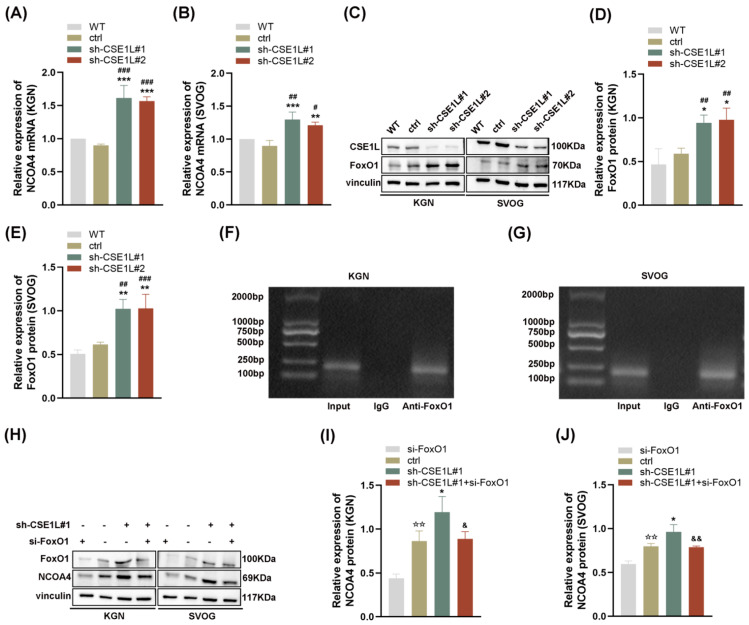
FoxO1 promoted the transcription of NCOA4. (**A**,**B**) NCOA4 mRNA expression in KGN and SVOG cells transfected with sh–CSE1L was detected qRT–PCR. (**C**) FoxO1 protein expression in KGN and SVOG cells transfected with sh–CSE1L was detected by Western blotting. (**D**,**E**) Quantification of band intensity of FoxO1/vinculin ratios in KGN and SVOG cells transfected with sh–CSE1L. (**F**,**G**) ChIP assay was conducted in KGN and SVOG cells. (**H**) NCOA4 protein expression in KGN and SVOG cells transfected with si–FOXO1 was detected by Western blotting. (**I**,**J**) Quantification of band intensity of NCOA4/vinculin ratios in KGN and SVOG cells transfected with si–FoxO1. N = 3 for each group. Data were expressed as mean ± SD. ^#^ *p* < 0.05 compared to WT, ^##^ *p* < 0.01 compared to WT, ^###^ *p* < 0.001 compared to WT. * *p* < 0.05 compared to ctrl, ** *p* < 0.01 compared to ctrl, *** *p* < 0.001 compared to ctrl. ^☆☆^ *p* < 0.01 compared to cells transfected with si–FoxO1. ^&^ *p* < 0.05 compared to cells transfected with sh–CSE1L#1, ^&&^ *p* < 0.01 compared to cells transfected with sh–CSE1L#1.

**Figure 8:**
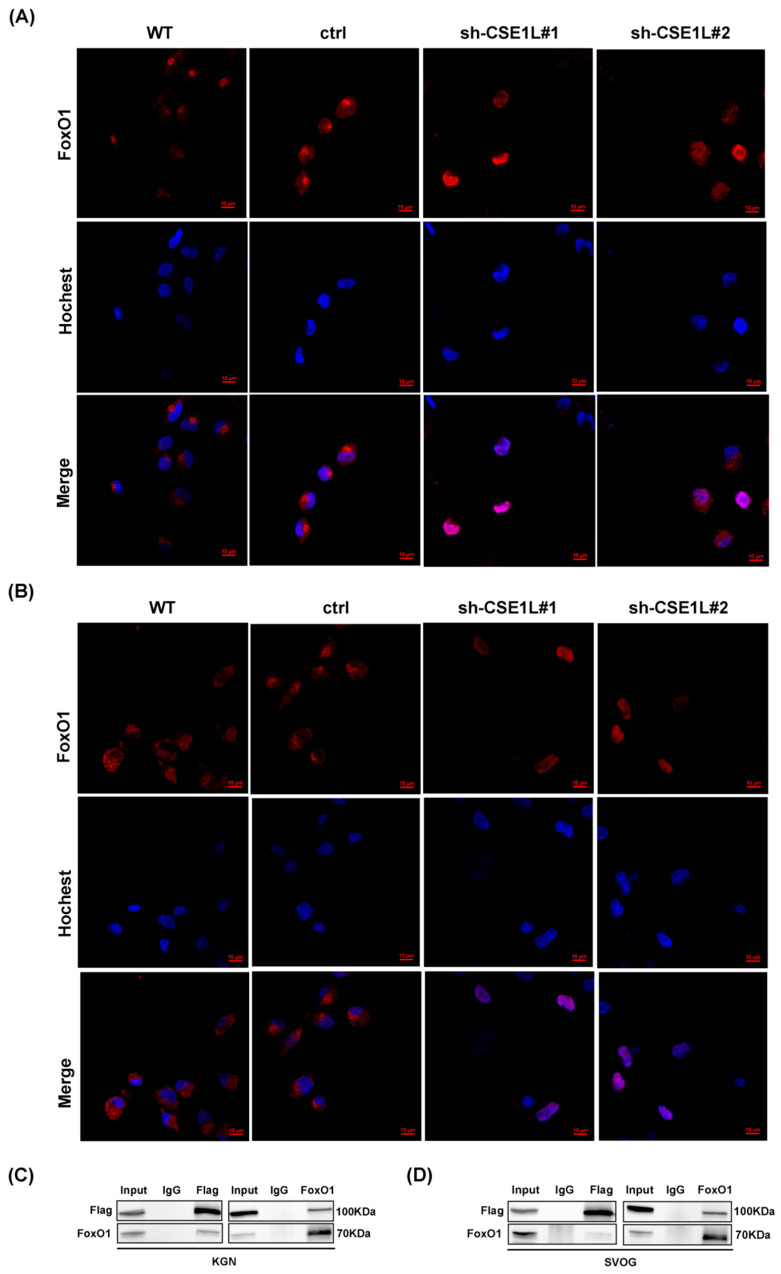
CSE1L participated in ferritinophagy via regulating the nucleocytoplasmic transport of FoxO1. (**A**,**B**) Localization of FoxO1 in KGN and SVOG cells transfected with sh–CSE1L. Immunofluorescence staining of FoxO1 (red) in cells were analyzed by confocal microscopy. Nuclei were counterstained with Hochest (blue). Scale bar: 10 µm. (**C**,**D**) Immunoprecipitation of the interaction between Flag–CSE1L and FoxO1 in KGN and SVOG cells. N = 3 for each group.

Given that NCOA4 was significantly increased in human granulosa cells transfected with sh–CSE1L, we speculated that the increased expression of NCOA4 was mainly caused by mRNA synthesis. Then, a qRT–PCR assay was performed. As expected, the NCOA4 mRNA level was significantly upregulated in cells transfected with sh–CSE1L (Figure 7A,B). To further investigate the underlying mechanism, the UCSC Genome Browser database was used to predict the potential transcription factors of NCOA4. Among them, FoxO1 aroused our interest. It is worth noting that FoxO1 is crucial to follicular development, granulosa cell proliferation, and autophagy [31]. Lin et al. [32] demonstrated that the overexpression of FoxO1 could induce granulosa cell apoptosis via upregulating FasL. Herein, we found that the expression of FoxO1 was upregulated by sh–CSE1L (Figure 7C–E). Next, the JASPAR database was used to predict the potential binding motif, and a ChIP assay was performed to verify it. As shown in Figure 7F,G, FoxO1 could bind to the NCOA4 promoter at the predicted binding motif (GAACACAGGA). To further investigate the effect of FoxO1 on NCOA4, si–FoxO1 was used. The results showed that si–FoxO1 could partially alleviate the increased NCOA4 induced by sh–CSE1L (Figure 7H–J). We actually know that only when the transcription factor enters the nucleus can it promote the transcription of the downstream targets. Given that CSE1L played an important role in maintaining cellular homeostasis via regulating the exportation of various cargoes from the nucleus to the cytoplasm [4], the nucleocytoplasmic transportation of FoxO1 was examined later. As expected, the loss of CSE1L indeed led to the excessive accumulation of FoxO1 in the nucleus (Figure 8A–B). Furthermore, coimmunoprecipitation (Co–IP) confirmed the direct interaction between CSE1L and FoxO1 (Figure 8C,D). Thus, these results demonstrate that CSE1L participated in ferritinophagy by affecting the nucleocytoplasmic transportation of FoxO1.

### 3.7. Inhibition of CSE1L in Cumulus Cell Monolayer Impeded Oocyte Maturation

**Figure 9:**
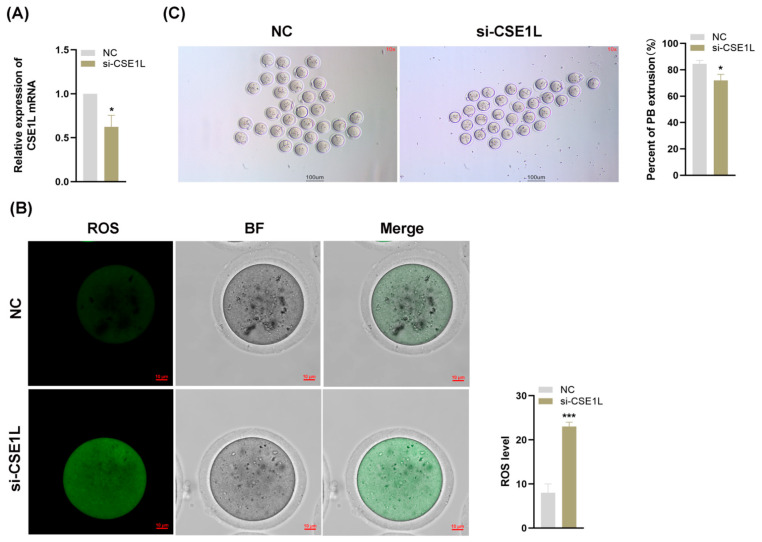
Inhibition of CSE1L in cumulus cell monolayer impeded oocyte maturation. (**A**) CSE1L mRNA expression in mice granulosa cells transfected with si–CSE1L was detected by qRT–PCR. (**B**) ROS levels of co–cultured oocytes. Scale bar: 10 µm. (**C**) The first polar body extrusion rate of co–cultured oocytes. Scale bar: 100 µm. N = 3 for each group (Each replication included at least 30 oocytes). Data were expressed as mean ± SD. * *p* < 0.05 compared to NC, *** *p* < 0.001 compared to NC.

Various studies have confirmed that the granulosa cell is essential to oocyte development [33]. Given that our study has demonstrated that the inhibition of CSE1L in human granulosa cells could induce ferroptosis, we supposed that the loss of CSE1L in cumulus cell monolayers might affect the development of oocytes. Then, we inhibited the expression of CSE1L in cumulus cell monolayers via si–CSE1L (Figure 9A) and constructed a co–culture system of cumulus cell monolayers and denuded oocytes. As shown in Figure 9B, inhibiting the expression of CSE1L in cumulus cell monolayers could increase ROS levels in the co–cultured oocytes. Moreover, compared with the control group, the first polar body extrusion rate of co–cultured oocytes was decreased, indicating that the oocyte maturation was impaired (Figure 9C).

## 4. Discussion

There were three main findings in this study. First, we identified a previously uncharacterized pathogenic homozygous variant of CSE1L (c. 1261T>C) in POI patients via targeted panel sequencing. Secondly, CSE1L was confirmed for the first time to participate in ferroptosis in human granulosa cells via nucleocytoplasmic transportation. Thirdly, the inhibition of CSE1L in cumulus cell monolayers could impede oocyte maturation, suggesting that CSE1L was essential to ovarian function.

The pathological mechanisms of POI included primordial follicle formation disorder, primordial follicle overactivation, and oocyte maturation disorder [34]. Actually, we know that the granulosa cell is essential to oocyte development. Granulosa cell dysfunction can initiate follicular atresia, which would further lead to POI. Herein, a co–culture system of cumulus cell monolayers and denuded oocytes was constructed to investigate whether the loss of CSE1L in granulosa cells had an impact on oocyte development. The results showed that si–CSE1L significantly impeded oocyte maturation via granulosa cells, which was similar to a retrospective review performed by Ni et al. [35]. The difference between the two studies was that Ni et al. [35] focused on endometriosis–related infertility rather than POI. They found that endometriosis–related infertile patients’ follicular fluid (EMFF) with an iron overload could induce ferritinophagy–mediated ferroptosis in granulosa cells [35]. Even worse, granulosa cells undergoing ferroptosis could further inhibit oocyte maturation by releasing exosomes [35]. Although some studies have confirmed that the free iron ion level is higher in the granulosa cells of cyclophosphamide–induced POI mice [36], the iron level in POI patients’ ovarian follicular fluid is unclear and needs to be further investigated.

The most studied mechanisms of granulosa cell dysfunction include steroidogenesis, apoptosis, autophagy, oxidative stress, mitochondrial dysfunction, and so on [37,38,39,40]. However, studies on the role of ferroptosis in granulosa cell dysfunction are rare. More surprisingly, the role of nucleocytoplasmic transportation in granulosa cell dysfunction has not been revealed up to now. In this study, RNA–seq suggested that CSE1L was closely associated with iron metabolism. Moreover, iron overload could lead to oxidative stress and lipid peroxidation in human granulosa cells, which would further induce ferroptosis. GPX4, famous as an antioxidant enzyme, plays an important role in inhibiting lipid peroxidation. Herein, we were surprised to find that sh–CSE1L could downregulate the expression of GPX4, while CSE1L overexpression of the lentivirus had the opposite effect. Overall, the loss of CSE1L aggravated lipid peroxidation by inhibiting the expression of GPX4.

NCOA4, widely known as a selective cargo receptor of ferritinophagy, is identified as an essential mechanism in mediating iron–dependent ferroptosis [41]. In this study, the expression of NCOA4 was increased in human granulosa cells transfected with sh–CSE1L. Then, the overexpressed NCOA4 bound to FTH1 and delivered FTH1 to the lysosome to degrade it in the presence of LC3 and ATG5/ATG7. Overall, the inhibition of CSE1L degraded FTH1 via NCOA4 to release more ferric iron, which might have further led to oxidative stress and lipid peroxidation. Sun et al. [42] found that the overexpressed NCOA4 was governed by the transcription factor JUN. Next, we used the UCSC Genome Browser database to predict the potential transcription factor. Among them, FoxO1 aroused our interest. Wang et al. [31] found that FoxO1 participated in the proliferation and autophagy of mice granulosa cells. Moreover, FoxO1 was involved in oocyte development [31]. Then, the potential binding motif was predicted by the JASPAR database and further verified by a ChIP assay. Moreover, si–FoxO1 was used to evaluate regulatory effects. The results showed that si–FoxO1 could partially alleviate the overexpressed NCOA4 induced by sh–CSE1L. In fact, we know FoxO1 can shuttle through the nucleus and cytoplasm via post–translational modifications. Only when a transcription factor enters the nucleus can it promote the transcription of the downstream targets. Given that CSE1L played an important role in nucleocytoplasmic transportation, we examined the localization of FoxO1. The results suggested that sh–CSE1L induced the excessive nuclear accumulation of FoxO1, which would further promote the transcription of NCOA4. Together, our study emphasizes the role of CSE1L in the development of POI, which might provide a more precise strategy for the treatment of POI.

## 5. Conclusions

In conclusion, a previously uncharacterized pathogenic homozygous variant of CSE1L was identified in POI patients. We first demonstrated that the inhibition of CSE1L could induce ferroptosis in human granulosa cells via nucleocytoplasmic transportation. Overall, these findings revealed an indispensable role of CSE1L in the development of POI and suggested ferroptosis inhibitors as potential therapeutic targets of POI. Certainly, our study had some limitations. Although we have investigated the role of CSE1L in human granulosa cells and mice oocytes, CSE1L conditional knockout mice are required to validate our results further.

## Data Availability

The data presented in this study are available on reasonable request from the corresponding author.

## Supplementary Materials

The following supporting information can be downloaded at https://www.mdpi.com/article/10.3390/antiox13080911/s1. Figure S1. The expression of CSE1L in human ovarian granulosa cells transfected with sh–CSE1L. Figure S2. GPX4 protein expression in KGN and SVOG cells transfected with CSE1L overexpression of the lentivirus was detected by Western blotting. Figure S3. Microphotographs from the immunofluorescence and TEM. Table S1. RNA sequence of sh–CSE1L. Table S2. Antibody lists. Table S3. Primer lists. Table S4. The ddG (kcal/mol) level in mutant CSE1L protein.
