# Supplementary material for: Chromosome Segregation–1–like Gene Participates in Ferroptosis in Human Ovarian Granulosa Cells via Nucleocytoplasmic Transport
Source: Antioxidants (Basel). 2024 Jul 28;13(8):911. doi: 10.3390/antiox13080911 (PMC11352033; doi:10.3390/antiox13080911)
Supplement: Supplementary file 1 [file antioxidants-13-00911-s001.zip › supplement tables.pdf]

Table S1. RNA sequence of sh-CSE1L

sh-CSE1L		Sequence
sh-CSE1L#1	CSE1L-i1F	gatccgCGCTGACAAGTATCTGTGAAAtcaagagTTTCACAGATACTTGTCAGCGtttttt
	CSE1L-i1R	aattaaaaaaCGCTGACAAGTATCTGTGAAActcttgaTTTCACAGATACTTGTCAGCGcg
sh-CSE1L#2	CSE1L-i2F	gatccgCCGTCTTCCTATATGGCCTTAtcaagagTAAGGCCATATAGGAAGACGGtttttt
	CSE1L-i2R	aattaaaaaaCCGTCTTCCTATATGGCCTTActcttgaTAAGGCCATATAGGAAGACGGcg

Table S2. Antibody lists

Antibody	Company	Cat No.
CSE1L	proteintech	67306-1-Ig
NCOA4	immunoway	YT0302
FoxO1	proteintech	18592-1-AP
FTH1	ABclonal	A1144
TF	immunoway	YT5194
TFR	immunoway	YT0775
GPX4	abcam	A1933
LC3	proteintech	14600-1-AP
ATG5	ABclonal	A19677
ATG7	ABclonal	A21895
IgG	Beyotime	A7028
beta-actin	proteintech	66009-1-Ig
vinculin	proteintech	66305-1-Ig
CoraLite594-conjugated Goat Anti-Mouse IgG (H+L)	proteintech	SA00013-3
CoraLite488-conjugated Goat Anti-Mouse IgG (H+L)	proteintech	SA00013-1
CoraLite594-conjugated Goat Anti-Rabbit IgG (H+L)	proteintech	SA00013-4
CoraLite488-conjugated Goat Anti-Rabbit IgG (H+L)	proteintech	SA00013-2

Table S3. Primer lists

Gene	Primer	Sequence
CSE1L	h-CSE1L-F	AGCAGCCCAGAGCAAATTCAGAAG
	h-CSE1L-R	GTCAAAGGCAAAGCAAAGGCATCC
CSE1L	m-CSE1L-F	ATGGAGTCCTTCGTACAGCG
	m-CSE1L-R	TCATTTGCATGGGTACTGCAC
NCOA4	h-NCOA4-F	GAGGTGTAGTGATGCACGGAG
	h-NCOA4-R	GACGGCTTATGCAACTGTGAA
NCOA4	h-NCOA4-F	GTAATCCCAGCTACTCAG
(chip)	h-NCOA4-R	ATCGCTCCACTGCACTCC

Table S4. The ddG (kcal/mol) level in mutant CSE1L protein

Gene	Variant Type	exon	Nucleotide change	Amino acid change	ddG (kcal/mol)
CSE1L	splicing mutation	exon 6	c. 477-3T>C	splicing	-
CSE1L	point mutation	exon 11	c. 1042A>G	p. I348V	3.142
CSE1L	point mutation	exon 13	c. 1261T>C	p. F421L	9.537
CSE1L	point mutation	exon 13	c. 1264C>T	p. P422S	5.644
CSE1L	point mutation	exon 5	c. 409C>T	p. R137C	3.234
