# Supplementary figures and images for: Chromosome Segregation–1–like Gene Participates in Ferroptosis in Human Ovarian Granulosa Cells via Nucleocytoplasmic Transport
Source: Antioxidants (Basel). 2024 Jul 28;13(8):911. doi: 10.3390/antiox13080911 (PMC11352033; doi:10.3390/antiox13080911)

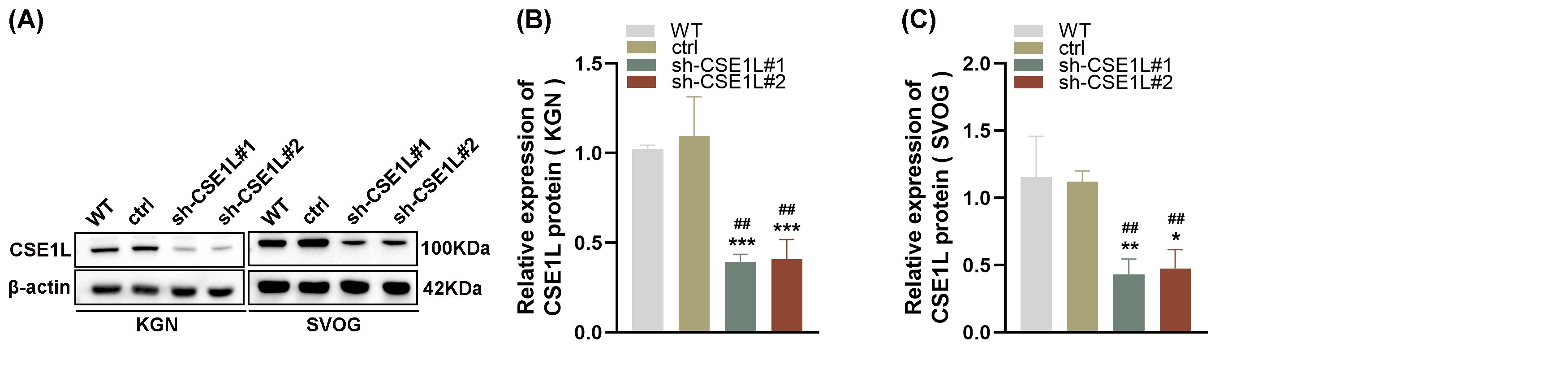

Supplement: Supplementary file 1 [file antioxidants-13-00911-s001.zip › Figure S1.tif]

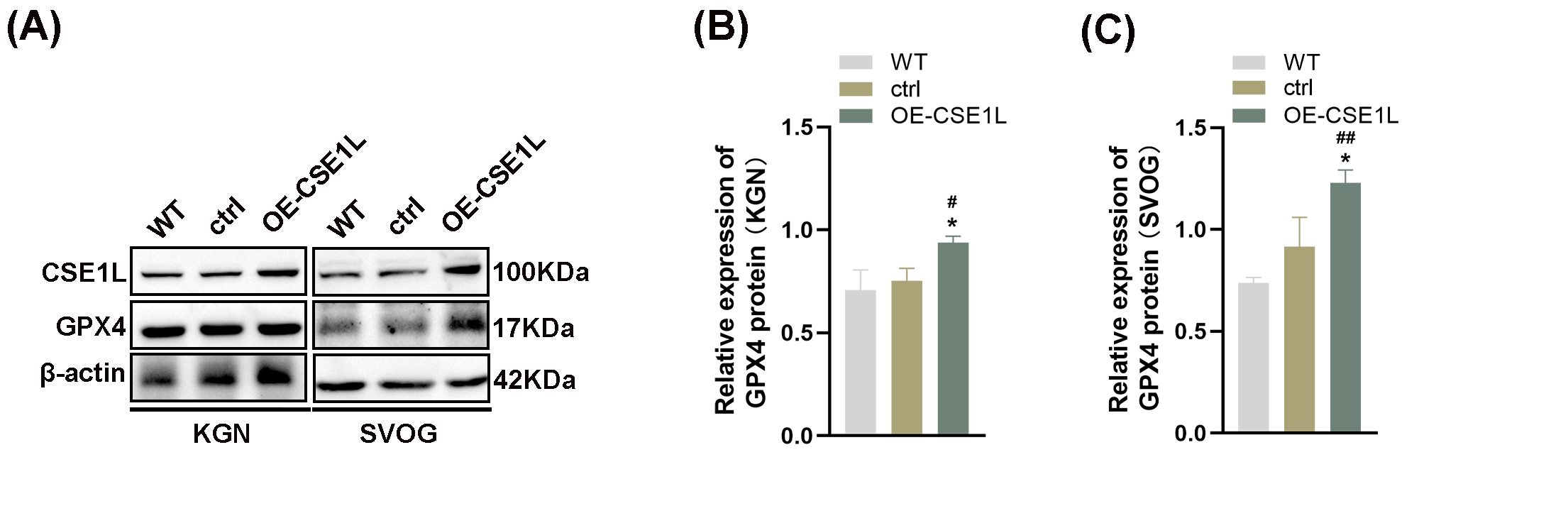

Supplement: Supplementary file 1 [file antioxidants-13-00911-s001.zip › Figure S2.tif]
